# Effectiveness of Serial Measurement of Differential Pressure in Closed Tibial Diaphyseal Fractures in Diagnosing Acute Compartment Syndrome using Whiteside’s Technique

**DOI:** 10.5704/MOJ.1603.001

**Published:** 2016-03

**Authors:** DR Ramprasath, V Thirunarayanan, J David, S Anbazhagan

**Affiliations:** Government Royapettah Hospital, Chennai, India

**Keywords:** Acute compartment syndrome, Whiteside’s technique, differential pressure, fasciotomy, tibial fractures

## Abstract

Acute Compartment Syndrome is a limb-threatening emergency and it occurs most commonly after fractures. The aim of our study is to find out the effectiveness of serial measurement of differential pressure in closed tibial diaphyseal fractures, in diagnosing acute compartment syndrome, using Whiteside’s technique. A total of 52 cases in the age group of 15 to 55 years admitted with closed fractures were studied for serial compartment pressure as well as serial differential pressure. Eight patients had persistent compartment pressure > 40mmHg, out of which only two patients had persistent differential pressure < 30mmHg and these two patients underwent fasciotomy. Thus, by measuring the compartment pressure serially and calculating differential pressure serially, acute compartment syndrome can be diagnosed or ruled out with higher precision, so that unnecessary fasciotomies can be avoided.

## Introduction

The average annual incidence of Acute Compartment Syndrome (ACS) is 3.1 per 1,00,000 population^1^, with incidence in males ten times that of females^1–5^. The reported incidence of ACS following tibial fracture varies from 2.7 to 11%^1,3,4^. Along with providing an improved patient outcome, an early diagnosis is associated with decreased indemnity risk forlegal claims^2,6^. Symptoms and signs of a compartment syndrome may be ambiguous, that, definite diagnosis cannot be made on clinical grounds alone^7–10^. Even though various techniques including saline infusion technique, slit catheter, wick catheter, Stryker STIC (Solid-state Transducer Intracompartmental) catheter and near infrared spectroscopy can be used to identify compartment pressure, we have measured Intra Compartmental Pressure (ICP) with Whiteside’s technique and formulated an easy and reliable protocol to diagnose ACS accurately. Among the above mentioned techniques, slit catheter and wick catheter techniques are inaccurate and out of vogue. Stryker STIC catheter, although accurate is very expensive and not available in all emergency departments. Near infrared spectroscopy is expensive and effectiveness has not been extensively studied.

Among the etiological factors, tibial diaphyseal fractures are the most common contributing cause for ACS. The aim of our study was to find out the effectiveness of serial measurement of differential pressure in closed tibial diaphyseal fractures, in diagnosing ACS, using Whiteside’s technique.

## Materials and Methods

This descriptive prospective study has been done involving 52 patients with closed tibial diaphyseal fracture in the age group of 15 to 55 years (Male:Female = 36:16) from December 2013 to May 2014 and follow up thereafter. The sample consisted of patients who presented to our emergency department consecutively with closed tibial diaphyseal fractures. Written consent was obtained from the patients for ethical clearance. Patients with compound fractures, multiple fractures, head injury, age < 15 years and > 55 years and known preexisting hypertension were excluded from the study.

Patient received in the emergency department were immediately subjected to head to foot survey and hemodynamic stabilization was done. Fractured limb was examined clinically for pain on passive stretching, tense compartment, pallor, paraesthesia, paralysis and pulselessness and the findings were recorded. Leg circumference was measured and compared with normal side. Eight patients who presented with tight cast underwent complete removal of cast. In all patients with ICP >40mmHg, limb elevation was avoided. Fracture was managed appropriately according to the need of the patient.

**Fig. 1:**
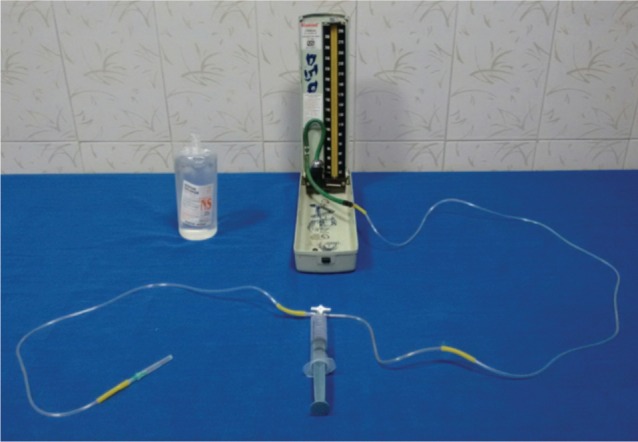
Whiteside’s Apparatus.

**Fig. 2:**
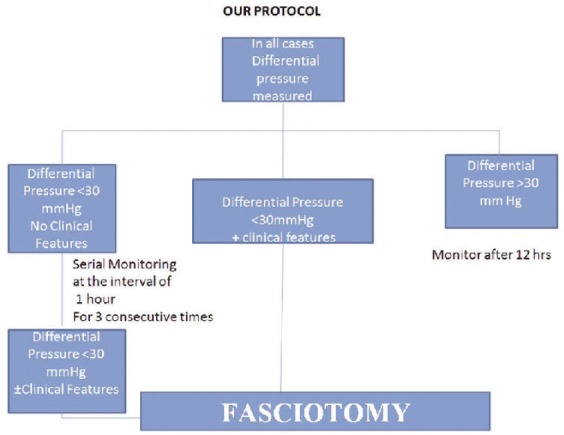
Treatment protocol.

We performed Whiteside’s technique of ICP measurement in the anterior compartment of leg within 5cm from the fracture site. Blood pressure was recorded in the upper limb simultaneously. Whiteside’s apparatus (Figure 1) was constructed using two IV sets, a 20 ml syringe, a 20 gauge needle, a blood pressure mercury manometer and a three way stopcock. One IV set was connected to 20 gauge needle and half filled with saline. The other IV set was connected to the mercury manometer. IV sets and 20 ml syringe with its plunger withdrawn up to 15ml mark were connected to stopcock. After preparing the leg, 20 gauge needle was inserted into the compartment keeping the apparatus at the level of limb. The plunger of the syringe was depressed slowly and mercury column will start rising in manometer. When the meniscus of saline changed from convex to flat in IV set, mercury column reading was noted in the manometer and recorded as ICP. Differential pressure (ΔP) was calculated using the formula : ΔP = Diastolic BP – ICP Serial monitoring (hourly) of ΔP and clinical features was done. The following protocol (Figure 2) was instituted in all the patients and results were recorded and statistical analysis was performed using descriptive analysis.

## Result

**Fig. 3:**
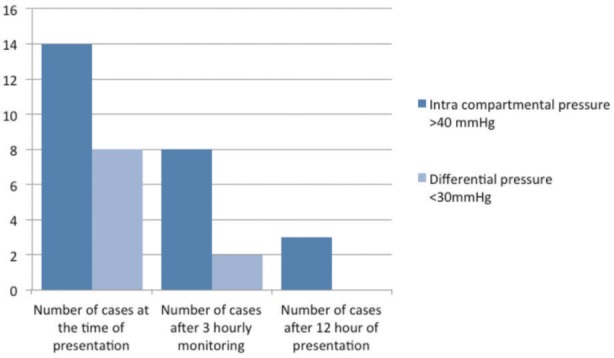
Bar chart comparing intracompartmental pressure and differential pressure.

There were 36 males and 16 females in our study. Mean of presentation’s time after injury was 3 hours (Figure 3). Mean of ICP at presentation was 36mmHg (Standard Deviation -10.36) and after 12 hours, it became 24mmHg (Table I). Mean of ΔP at presentation was 42mmHg (Standard Deviation-11) and it became 52mmHg after 12 hours of monitoring.

**Table I:** Distribution of cases in ICP and ΔP category

Parameters	No of cases at the time of presentation	No of cases after 3 consecutive hourly monitoring	No of cases after 12hrs of presentation
Compartment pressure ( > 40 mmHg)	14 (27%)	8 (15%)	3 (6%)
Compartment pressure ( <40 mmHg )	38 (73%)	44 (84%)	47 (90%)
Differential pressure ( < 30 mmHg )	8 (15%)	2 (4%)	0
Differential pressure (>30 mmHg)	44 (84%)	50 (96%)	50 (96%)

Two patients who had ΔP < 30mmHg on serial monitoring underwent fasciotomy. The eight patients in whom tight cast was removed subsequently showed ΔP >30mmHg on serial monitoring. The follow up of all patients (except those who underwent fasciotomy) was uneventful at the end of six months.

## Discussion

Although clinical signs; like pain on passive stretch, pallor, tense compartment, help in diagnosing ACS, they may be absent in some cases, indicating poor sensitivity. The clinical findings of pain, pulselessness etc for diagnosing ACS was low at 13% to 19% and specificity was 97%, meaning all the clinical signs are better at excluding than confirming a diagnosis^2–4,11,12^. Badhe *et al*reported four cases of ACS in which any form of pain was absent, with diagnosis confirmed on subsequent fasciotomy^2,13^.

The normal ICP of healthy muscle is 10mmHg^1,2,14^. It is now recognized that a patient’s tolerance to the ICP is dependent on perfusion or the systemic blood pressure^2,15–19^. There is now experimental and clinical work suggesting a ΔP of less than or equal to 30mmHg should be considered diagnostic 2,15,20-22. The use of a differential pressure of <30 mmHg as a threshold for fasciotomy lead to no missed cases of ACS, no unnecessary fasciotomies and no complications in any patients^1,2,15^. In our study, 75% of patients who had ICP > 40mmHg did not undergo fasciotomy, since serial ΔP monitoring was instituted in these patients.

Studies have demonstrated that the duration of pressure elevation is fully as important in the production of neuromuscular deficits as is the magnitude of pressure elevation. Pressures that are benign for a few hours may be detrimental if allowed to persist for longer periods. Thus, serial monitoring of tissue pressure provides clinically useful information as to the trend of ICP^8,24,25^.

Muscles tolerate 4 hours of ischemia well, but by 6 hours the result is uncertain, after 8 hours, the damage is irreversible ^26^, hence the early diagnosis becomes important. In the leg, the anterior compartment is routinely used for pressure monitoring, as it is the most frequently affected and accessible^2,15,27^. Our study, as well as literature, supports the fact that any limb showing signs of a compartment syndrome is freed from circumferential dressings and is placed at the level of the heart to ensure that local blood pressure is not compromised by elevation of the extremity^7,28,29^.

The Stryker STIC catheter is a device specifically designed to measure ICP. Because it is expensive, not all medical facilities may have access to this device. Alternative ICP measurement methods to the Stryker device include the IV pump method and the Whiteside’s technique^30^. Among these, Whiteside’s technique is inexpensive, easily available and easy to assemble.

## Conclusion

The use of differential pressure as a parameter particularly when measured serially helps in accurate decision making in either performing or avoiding fasciotomies. Being an easier parameter to measure, this method seems to be far better in making decision than methods based on ICP measurement alone. Whiteside’s technique which is more or less nearer to accuracy when compared to Stryker STIC catheter, and at the same time is cheap and easily available, will help more institutions to diagnose and treat ACS effectively. It also protects the treating team against litigations in the court of law.
